# Nitrate respiration occurs throughout the depth of mucoid and non-mucoid *Pseudomonas aeruginosa* submerged agar colony biofilms including the oxic zone

**DOI:** 10.1038/s41598-022-11957-4

**Published:** 2022-05-20

**Authors:** Carsten Ulrich Schwermer, Dirk de Beer, Paul Stoodley

**Affiliations:** 1 Norwegian Institute for Water Research (NIVA), Gaustadalléen 21, 0349 Oslo, Norway; 2 Max Planck Institute for Marine Microbiology (MPI), Celsiusstrasse 1, 28359 Bremen, Germany; 3 Department of Microbial Infection and Immunity, Department of Orthopaedics, The Ohio State University, Biomedical Research Tower (BRT), 716, 460 W 12th Ave, Columbus, OH 43210 USA; 4 National Centre for Advanced Tribology at Southampton (nCATS) and National Biofilm Innovation Centre (NBIC), Mechanical Engineering, University of Southampton, Southampton, UK

**Keywords:** Infectious diseases, Biological techniques, Microbiology

## Abstract

*Pseudomonas aeruginosa* is an opportunistic pathogen and well characterized biofilm former. *P. aeruginosa* forms strong oxygen gradients inside biofilms due to rapid oxygen respiration in the top layers and the poor solubility of oxygen coupled with diffusion limited transport. Transcriptomic evidence from in vitro and ex vivo sampling suggests that denitrification is occurring in biofilms in ostensibly oxic environments. It is hypothesized that in the presence of nitrate there is stratification with aerobic respiration occurring in the outer oxic layer and denitrification in the lower anoxic zone. We used submerged agar colony biofilms grown from mucoid (FRD1) and non-mucoid (PAO1) strains to simultaneously measure depth microprofiles of oxygen and nitrous oxide in the same colony with microelectrodes. Oxygen respiration occurred at the top of the colony as expected but denitrification occurred throughout the entire depth, even in the oxic region. Local denitrification rates were highly variable suggesting heterogenous metabolic activity within the colony. We also assessed the short-term influence of tobramycin on aerobic respiration within a PAO1 colony. Although there was an immediate reduction in respiration it was never completely arrested over a 2 h period. On tobramycin removal the oxygen gradient steadily reestablished, demonstrating immediate recovery of metabolic activity.

## Introduction

*Pseudomonas aeruginosa* is a Gram-negative opportunistic pathogen and is a well characterized biofilm former. Biofilm formation by this organism is a recognized virulence factor in chronic infections such as occurring in the cystic fibrosis (CF) lung^1^ and unhealing wounds^2^. Anaerobic nitrate (NO_3_^−^) respiration (denitrification), even in an ostensibly physiologically aerobic environment, such as the lung, has been found to be a relevant factor in the pathogenesis of this organism^3,4^. It is thought that hypoxic conditions in the mucus of the CF lung are caused by greater consumption of oxygen by the underlying CF epithelial cells^5^. However, it is well established that biofilms formed from aerobic or facultative aerobes, such as *P. aeruginosa*, can form strong oxygen gradients due to the rate of aerobic respiration near the surface of the biofilm consuming oxygen at a greater rate than oxygen can diffuse in from the overlying fluid^6^. Expression levels of genes of enzymes involved in denitrification [i.e., 2NO_3_^−^ → 2NO_2_^−^ → 2NO^−^ → N_2_O → N_2_ by nitrate reductase (NAR), nitrite reductase (NIR), nitrous oxide reductase (NOR), and nitrous oxide reductase (NOS)^7^ were found to be upexpressed in anaerobically grown *P. aeruginosa* biofilms. Also, the outer membrane protein OprF, which is associated with anaerobic growth, was found in biofilms collected from *P. aeruginosa* infected clinical samples of CF sputum^8^, providing evidence for anaerobic growth in the CF lung. In addition, denitrification in *P. aeruginosa* was demonstrated in vitro under hypoxia and physiologically relevant NO_3_^−^ concentrations (400 µM) of the CF lung by RT-PCR, N_2_O microelectrodes, GFP reporter constructs and proteomic^4,9^. While these studies provide compelling evidence that denitrification metabolism by *P. aeruginosa* occurs under hypoxia and are likely occurring in the CF lung, they do not provide data on spatial distribution of anaerobic activity within the biofilm. It is generally thought that the outside layer of the biofilm adjacent to the oxygenated fluid would be dominated by aerobic respiration, while the less energetically favorable anaerobic respiration or fermentation would occur in the interior of the biofilm, resulting in species and phenotypic stratification in a mixed species biofilm^10^. Unlike oxygen, which is sparingly soluble in water (i.e. 8.3 mg/L, at 25 °C, 0.9% salinity and 1 bar), NO_3_^−^ as an alternative electron acceptor is over a thousand times more soluble and thus may be expected to fully penetrate the biofilm. We hypothesized that in the presence of both oxygen and NO_3_^−^, aerobic respiration would occur in the upper layer of the biofilm and that denitrification would occur primarily below this layer in the anoxic region created by oxygen depletion. Previously microelectrodes for different ions and molecules have been used to create concentration depth profiles in biofilms to understand the distribution of different metabolic processes and to infer the stratification of different physiological species in biofilms to assess community interactions and local and overall metabolic activities^11^. Here we used a simple in vitro model to simulate biofilms growing on soft surfaces where colonies of mucoid (FRD1) and non-mucoid (PAO1) *P. aeruginosa* strains were grown on agar plates and submerged in a glass aquarium reservoir. The model was specifically designed to allow the simultaneous measurement of vertical high-resolution microprofiles of dissolved oxygen and nitrous oxide (N_2_O) after spiking with NO_3_^−^ by microelectrodes, which were introduced at the same depth of the biofilm, angled in from different sides. The corresponding NO_3_^−^ microprofile was calculated from the stoichiometry of N_2_O production.

## Materials and methods

### Bacterial strains, growth conditions and experimental set-up

**Figure 1:**
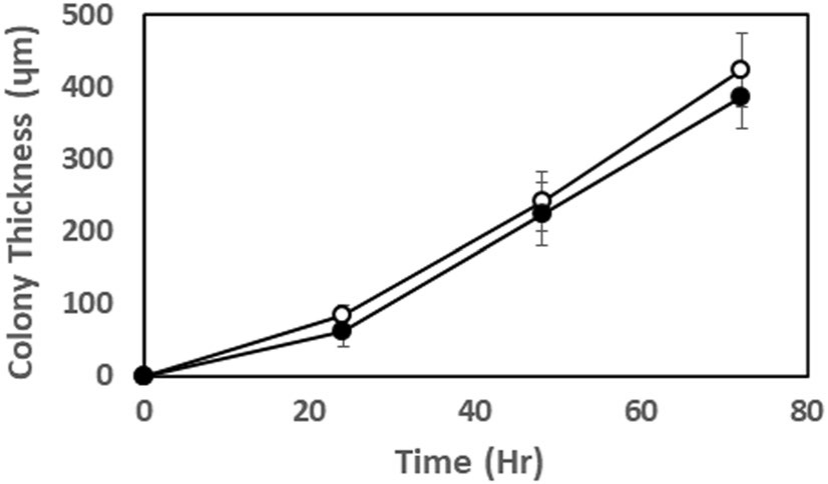
Colony thickness as function of time for PAO1 (open circles) and FRD1 (black circles). Mean and 1. STD of five measurements from different colonies on the same petri plate.

We used *P. aeruginosa* PAO1, a non-mucoid wound isolate^12^, and FRD1 a mucoid cystic fibrosis isolate^13^. Frozen stock cultures were streaked out on brain heart infusion agar BHI (Difco 241830) and incubated for 24 h at room temperature (24 °C) for three days. BHI has been noted to suppress mucoidy in *P. aeruginosa*^14^, allowing distinct colonies of FRD1 to present on the agar. The average thickness of the biofilm colonies on a dedicated set of plates was measured daily by microscopy by focusing on the top of the colonies and the adjacent bare agar and noting the distance travelled using the scale on the focusing knob (Fig. 1). The plates were then transferred to a square glass sided (approximately 14 × 14 × 7 cm in dimensions) aquarium reservoir which was gently filled with 0.75 L of minimal salts media (MSM supplemented with glucose in g/L: 0.7 K_2_HPO_4_, 0.3 KH_2_PO_4_, 0.01 NH_4_SO_4_ MgSO_4_ × 7H_2_O, 0.4 glucose, all from Sigma-Aldrich) to submerge the biofilm. The pH was adjusted to 7.3 with NaOH. The Petri plate was weighed down by wrapping with a flexible plastic-coated lead flask weight. A recirculating pump was used to generate water flow to allow mixing in the reservoir and air was introduced through an air stone to maintain oxygen saturation in the water phase. Floating balls (Allplas, Capricorn Chemicals Corp., Secaucus, NY) covered the water surface to minimize gas exchange with the atmosphere. When the MSM was added to the reservoir there was no immediate visible disruption to the colonies (Fig. 1C,D); however, in preliminary experiments after 3 or 4 h we observed dissolution of the colonies with a concomitant clouding of the media, thus we performed our experiments within limited time frames prior to dissolution of the colonies evidence by clouding at the colony surface from observation using the dissecting microscope.

### Microelectrodes and calibration

Dissolved oxygen (DO) and nitrous oxide (N_2_O) microelectrodes with tip diameters of approximately 10 µm were fabricated at the Max Planck Institute for Marine Microbiology as previously described^15,16^. The build-up of N_2_O on the addition of NO_3_^−^ is indicative of denitrification according to the reaction series. The microelectrodes were connected to a picoammeter and the signal in pA were recorded by a data acquisition system (model DAQCard AI16XE50; National Instruments, Austin, TX) on a laptop. DO electrodes were calibrated in saturated (air sparged) and anoxic (N_2_ sparged) phosphate buffered water (MSM). DO concentration at 25 °C and 1 ATM is 8.33 mg/L (260 µM). The N_2_O electrodes were calibrated with MSM solutions at 0, 100, 200, 300, 400 µM N_2_O by first saturating the MSM with N_2_O gas and then by dilution. Since the N_2_O in solution slowly escape to the atmosphere, the concentration can only be considered constant for a few minutes, thus the calibration was performed quickly. There was a linear relationship between measured pA and N_2_O concentration (Supplementary Fig. 1). However, we did see some variation in the slope and the intercept in calibrations taken over multiple days. The standard deviation of the slope as a percent of the mean of 5 calibration curves taken at 5 different days was 22% but the y-axis intercept (the signal at zero N_2_O concentration) was 117%. The signal was relatively stable over the measurement of a single profile (approximately 11 min) (Supplementary Fig. 2) with the SD as a percent of the mean was 1.07%. However, we did observe baseline drift occurring over longer time periods. To correct for this we used subtracted the baseline from the signal in the bulk fluid where N_2_O was expected to be zero and DO was expected to be saturated, also indicated by vertical profiles outside of the diffusion boundary layer. In some cases when we repositioned the microelectrode between profiles there were differences in the surface of the colony relative to our initial positioning. To account for these we adjusted the profile depth so that the linear part of the DO or N_2_O profile (reflecting the diffusion boundary layer) was at the approximate surface of the biofilm (within an estimated 25 µm).

While some bacterial species reduce NO_3_^−^ to N_2_O, if there is complete reduction to N_2_ gas, N_2_O is consumed and consequently denitrification activity may be underestimated. *Pseudomonas aeruginosa* is not known to produce N_2_ gas but as a control to check this with our strains, acetylene (10% of a saturated solution in the MSM achieved by sparging with pure acetylene gas), which prevents the reduction of N_2_O to N_2_^17^, was added after a NO_3_^-^ spike (1.2 µM) to an FRD1 biofilm colony and no difference in N_2_O concentration was seen (Supplementary Fig. 3).

### Microelectrode measurements

**Figure 2:**
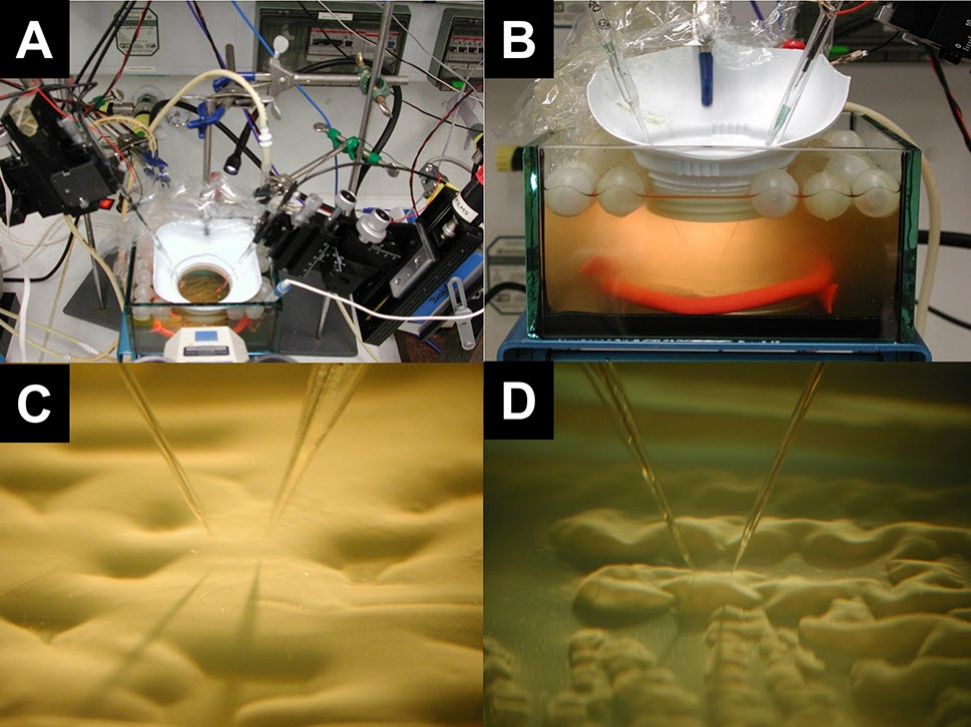
Experimental set-up for ex vivo agar plate colony biofilm growth modelling. (**A**) Reservoir containing the petri plate with agar colony biofilms showing the positioning of the DO and N_2_O microelectrodes (left and right) and the reference electrode (center). (**B**) Closer view from the side showing the electrodes in the reservoir. (**C**,**D**) The two microelectrodes just touching the surface of colonies grown on two different plates. The surface of the colony biofilm was identified when the shadows cast by the electrodes co-localized with the tips of the microelectrodes. An even flow of growth medium was supplied above the colonies by a pump (invisible).

The DO and N_2_O microelectrodes were inserted into the reservoir through a circular “window” fabricated from the top of a plastic wide mouth container using a micromanipulator (model MM33; Maerzhaeuser, Wetzlar, Germany) with a motor-controlled z-axis stepper (model VT-80; Micos, Eschbach, Germany). Positioning during vertical profiling and data acquisition were controlled by using custom-made software. The electrodes were simultaneously lowered onto the surface of the same colony biofilm while being observed with a dissecting microscope under illumination by a gooseneck lamp (Schott). The approximate surface of the colony biofilm was identified by the point at which the tips of the microelectrodes and their shadows cast by the gooseneck lamp co-localized (Fig. 2). A more precise identification of the colony-bulk fluid interface was made by noting a rapid response of the DO microelectrode, which was between 25 and 50 µm deeper than the visual estimate. The surface of the colony biofilm was designated as depth 0 µm. Distances above the interface in the bulk fluid were designated as negative and below into the colony biofilm as positive. Profiles of DO and N_2_O outside and within the colonies were generated generally from 500 µm above the surface to a depth of 500 µm into the biofilm at 25 µm steps. The stepper motor travel step distance to achieve 25 µm steps at the tips of the microelectrodes was calculated from the angle of insertion by simple geometry. The reservoir system and microelectrodes entering a colony biofilm are shown in Fig. 2. Denitrification was induced by spiking the reservoir with a hundred times concentrated 10 g/L NaNO_3_-stock solution to achieve a final concentration of 0.1 g/L (1.2 mM) of NO_3_^-^. DO and N_2_O profiles were measured repeatedly at the same locations until steady state was reached, which is an important condition for flux calculations. To calculate aerobic respiration and denitrification rates and activities as a function of depth two sets of profiles were measured from different colonies on the same plate. The measurements are therefore suitable to compare these activities in almost the same location but do not provide information on variability between biofilm colonies grown on different plates, or indeed, cannot be used to make conclusions regarding differences between colonies on the same plate.

### Influence of oxic, oxygen supersaturation and anoxic conditions on aerobic respiration and denitrification

To assess the influence of the head space gas composition we took simultaneous measurements of DO and N_2_O in a PAO1 biofilm colony after sparging with air, then switching to pure oxygen, then returning to air and finally with pure nitrogen. Unfortunately, due to equipment issues we were not able to measure a DO profile when the system was sparged with pure N_2_.

### Short term exposure to tobramycin

Since anoxic conditions within *P. aeruginosa* biofilms have been associated with tolerance to many antibiotics including aminoglycosides, quinolones and β-lactams^18^ through either dormancy or the production of phezanines^6^ in the anoxic regions, we assessed the shorth term exposure of tobramycin on aerobic respiration at 100 µm depth in a PAO1 biofilm colony. A profile between − 800 µm to 400 µm depth in 20 µm steps into the biofilm was taken 1.0 h prior to tobramycin addition at 10 µg/mL in MSM (approximately 5 times the minimum inhibitory concentration, MIC). The DO microelectrode was then parked at 100 µm depth and the influence on aerobic respiration activity measured as decreasing DO concentration. The DO was measured for 1.0 h prior to tobramycin addition to establish the baseline aerobic activity at that depth in the biofilm and then for a further 2.0 h after tobramycin addition. A second profile was taken at 2.5 h after tobramycin addition. The reservoir was then exchanged with MSM with no antibiotic and measurements continued for a further 2.5 h. We note that the system was continually sparged with air to maintain oxygen saturation over the course of the experiment.

### Dissolved oxygen (DO) and NO_3_^−^ flux calculations

Since denitrification from NO_3_^−^ to N_2_O proceeds in a stoichiometry of 1:1, we calculated the NO_3_^−^ concentration at each level in the profile by subtracting the molar concentration consumed in the production of the measured concentration of N_2_O from the starting NO_3_^−^ concentration of 1.2 mM in the bulk fluid. We calculated the flux of DO and NO_3_^−^ consumption by the colonies as well as a function of depth to determine where aerobic respiration and denitrification were predominantly occurring in the agar colony biofilm. At steady state the diffusive transport of solutes through the diffusive boundary layer (DBL) in the bulk fluid adjacent to the colony is proportional to the concentration gradient in the DBL according to Fick’s first law of diffusion: J = D_c_ (∂c/∂x), where J is the flux (nmol cm^−2^ s^−1^), D_c_ is the molecular diffusion coefficient (cm^2^ s^−1^), and ∂c/∂x, is the concentration gradient (nmol cm^−4^) at the colony biofilm bulk fluid-interface^19,20^. Assuming a D_c_ in water of 2.36 × 10^–5^ cm^2^ s^−1^ for oxygen and of 1.84 × 10^–5^ cm^2^ s^−1^ for NO_3_^−^ at 24 °C^21,22^, and correcting for a reduced effective D_c_ (D_eff_) in the biofilm of approximately 58% and 68% of that in water as estimated for both DO and NO_3_^−^ respectively^23^, areal uptake rates can be calculated that represent the total biofilm by the colony. Local activity of aerobic respiration and denitrification at different depths in the colony biofilm was found from the slope of the profiles within the biofilm colonies using a modified version of Fick’s second law of diffusion (assuming diffusion is the dominant transport process within the biofilm): D_eff_ (∂^2^c/∂x^2^) = r, where ∂^2^c/∂x^2^ is the concentration gradient between each step and r is the local conversion rate (mol cm^−3^ s^−1^)^24^. The overall fluxes of DO and NO_3_^−^ into the colonies were calculated from the concentration–distance slope of the first three points at the surface (0–50 µm) and multiplying by the assumed D_eff_ as previously described^20^. While we produced many profiles showing similar trends, we chose overall activity fluxes that were calculated from two representative profiles from each strain taken at different biofilm colonies from the same plate. Therefore, these values can be used to compare the relative local uptake rates of NO_3_^−^ and DO associated with those particular colonies, but do not account for biofilm heterogeneity and are therefore not suitable for the calculation of average fluxes over the total biofilm surface as previously discussed^20^. Similarly, these data are not statistically robust enough to draw strong conclusions regarding differences of fluxes between the two strains.

## Results

**Figure 3:**
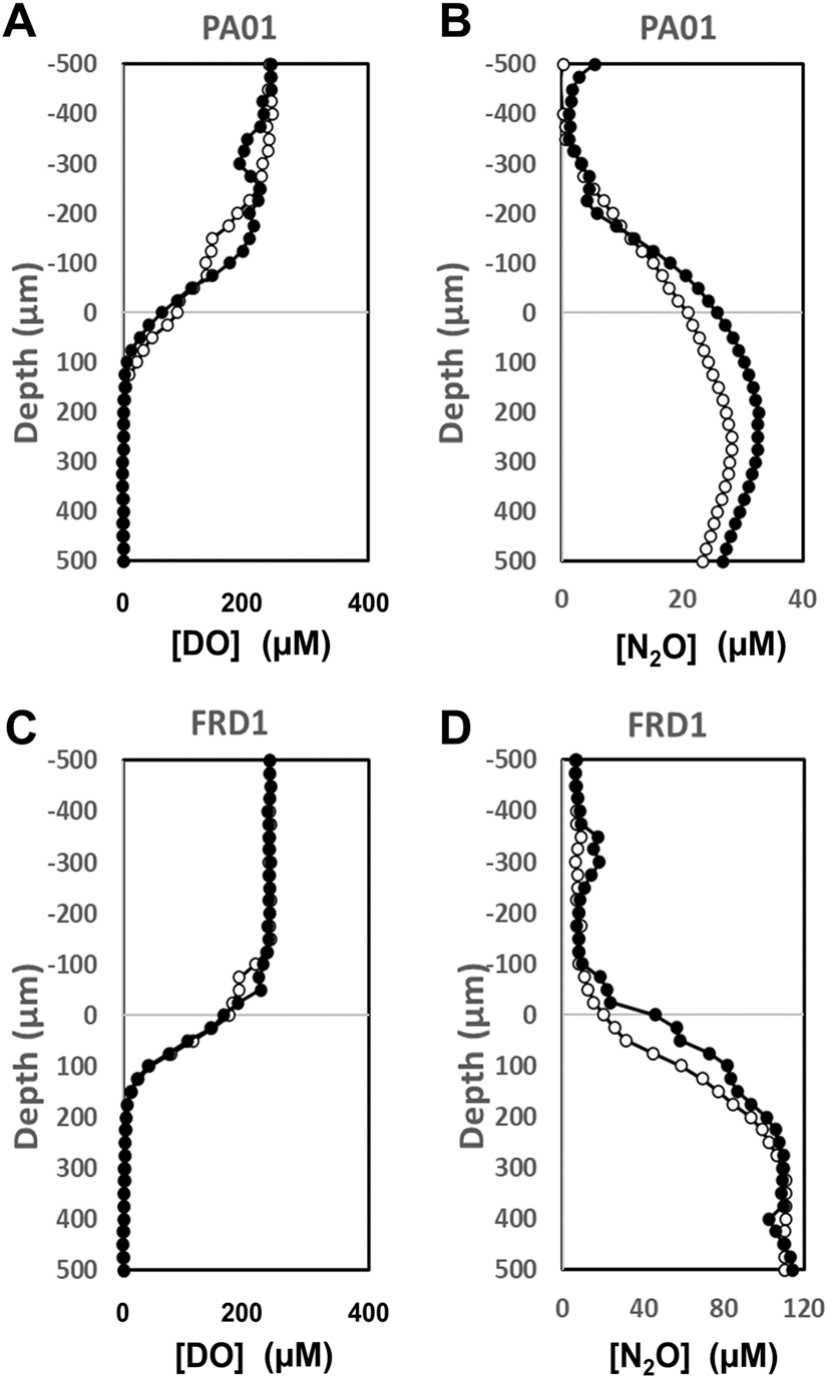
DO (**A**,**B**) and N_2_O (**C**,**D**) profiles in PAO1 and FRD1 agar colony biofilms measured in two different colonies (n = 2) on the same petri plate (open and closed symbols) after the addition of 1.2 mM NaNO_3_. The bulk liquid-colony biofilm interface is presented by 0 µm depth. While positive values present the colony biofilm body, negative values present the liquid phase.

**Figure 4:**
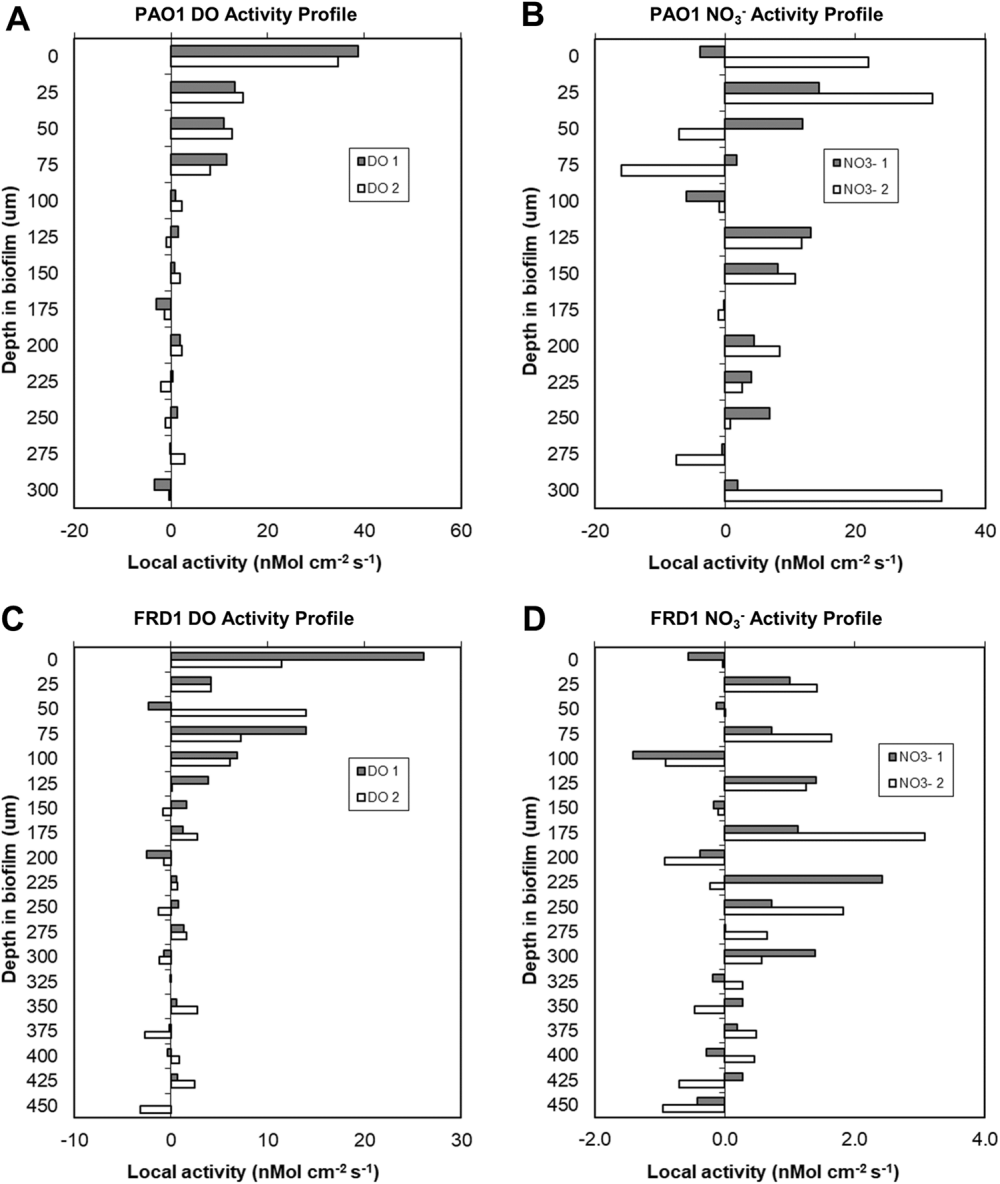
Local activity of aerobic respiration and denitrification (calculated from NO_3_^−^ consumption based on N_2_O measurements) as a function of depth in PAO1 and FRD1 colony biofilms measured in two different colonies on the same plate (light and dark bars) after the addition of 1.2 mM NaNO_3_. The bulk liquid-colony biofilm interface is represented at 0 µm biofilm depth. Positive values on the x-axis of DO and NO_3_ profiles show aerobic respiration and denitrification, respectively. Calculations are based on and correspond to the concentration profiles shown in Fig. 3.

**Figure 5:**
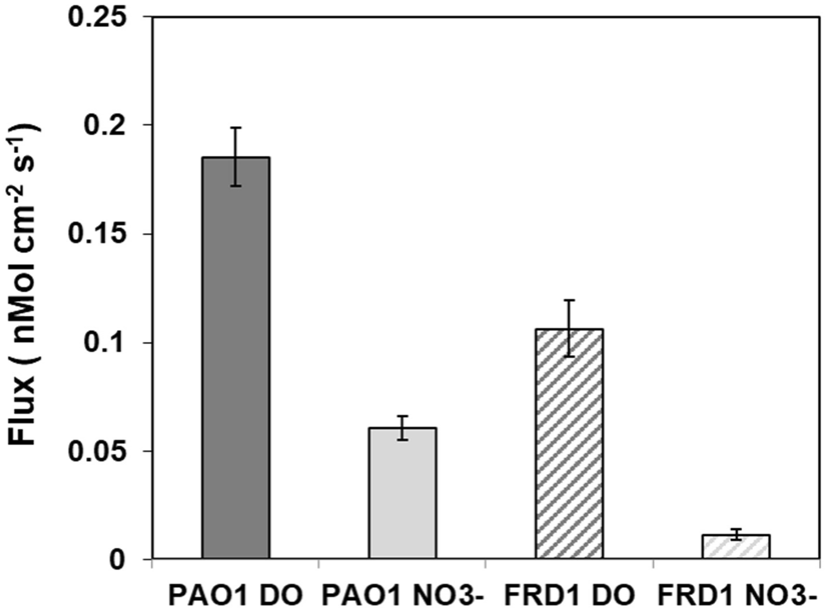
Local areal uptake rate of DO and NO_3_^−^ measured for in PAO1 and FRD1 colony biofilms on the same plate (light and dark bars indicate data from the different profiles) after the addition of 1.2 mM NaNO_3_.

After 72 h incubation at room temperature, both PAO1 and FRD1 colonies had grown to approximately 400 µm thickness (Fig. 1). In both PAO1 and FRD1 sharp DO gradients developed at the colony biofilm bulk liquid interface and the colonies became anoxic at approximately 100 µm (PAO1) and 150 µm (FRD1) depths (Fig. 3A,B), with the maximum N_2_O concentration at steady state being at approximately 200 µm (PAO1) and 250 µm (FRD1) depth (Fig. 3C,D). In BHI alone there was no detection of N_2_O production until denitrification was stimulated by spiking with 1.2 mM NO_3_^-^ (Supplementary Figs. 2, 3), however, for the PAO1 colony when we ran a control we did see N_2_O production in BHI alone, but this was increased when spiked with 1.2 mM NO_3_^−^ (Supplementary Fig. 4). Differences between the two responses might be due to metabolic differences between the two strains under our testing conditions. Also metabolic activity while we were setting up the measurements, which although we tried to minimize, may have led to changing the nutritional conditions. Local activity profiles showed that the greatest/DO consumption was occurring in the surface layers of the biofilm colonies and steadily decreased with depth until there was little or no DO respiration ≤ 100 µm (Fig. 4A,B). However, the calculated NO_3_^−^ consumption showed that denitrification was more uniform throughout the depth of the colony and was occurring in the surface layers of the biofilm colonies in the oxic zone (Fig. 4C,D). For both strains the overall consumption rate of DO was much greater than that of NO_3_^−^ by a factor of 3 and 22 for PAO1 and FRD1, respectively (Fig. 5).

### Influence of oxic and anoxic conditions on aerobic respiration and denitrification

**Figure 6:**
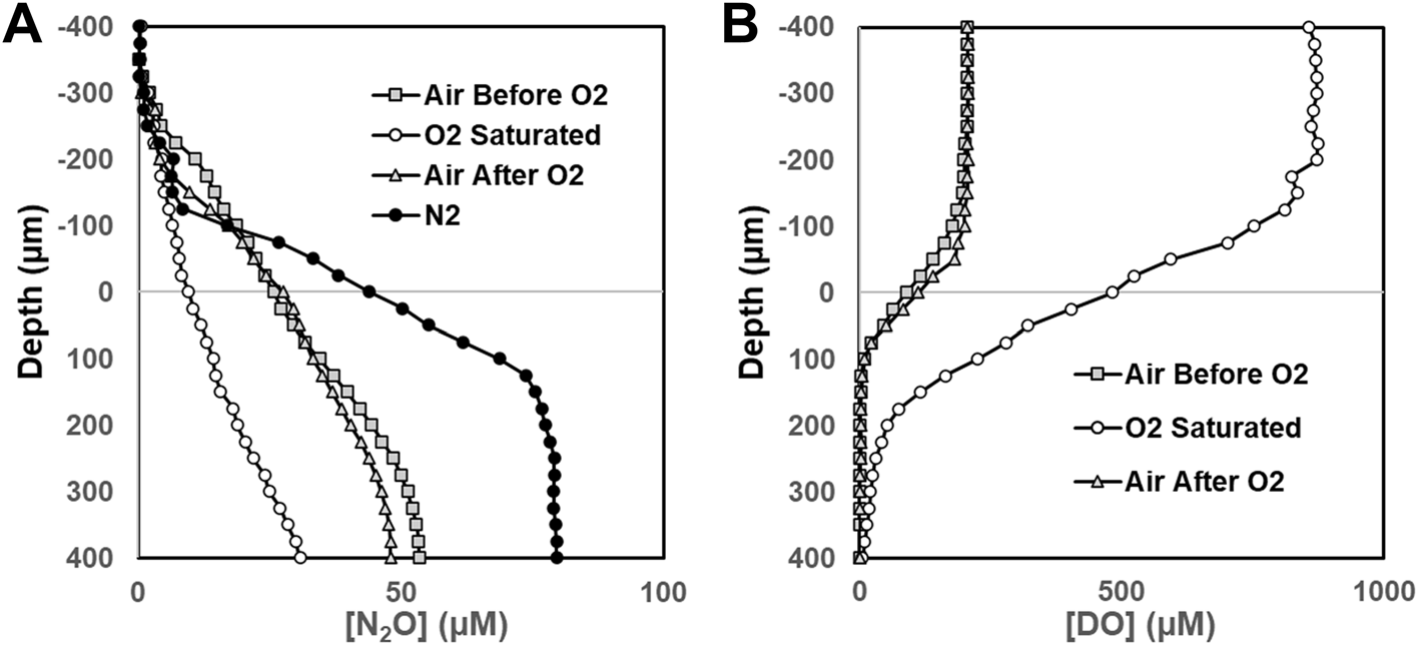
Oxygen supersaturation suppresses denitrification but stimulates aerobic respiration and anoxic conditions stimulate denitrification in a PAO1 biofilm colony. Profiles taken under sparging with air (air before O_2_), 10 min after sparging with pure oxygen (O_2_ saturated), 10 min after returning to sparging with air (air after O_2_) and 80 min after sparging with pure nitrogen (N_2_). (**A**) N_2_O profiles and (**B**) DO profiles.

**Figure 7:**
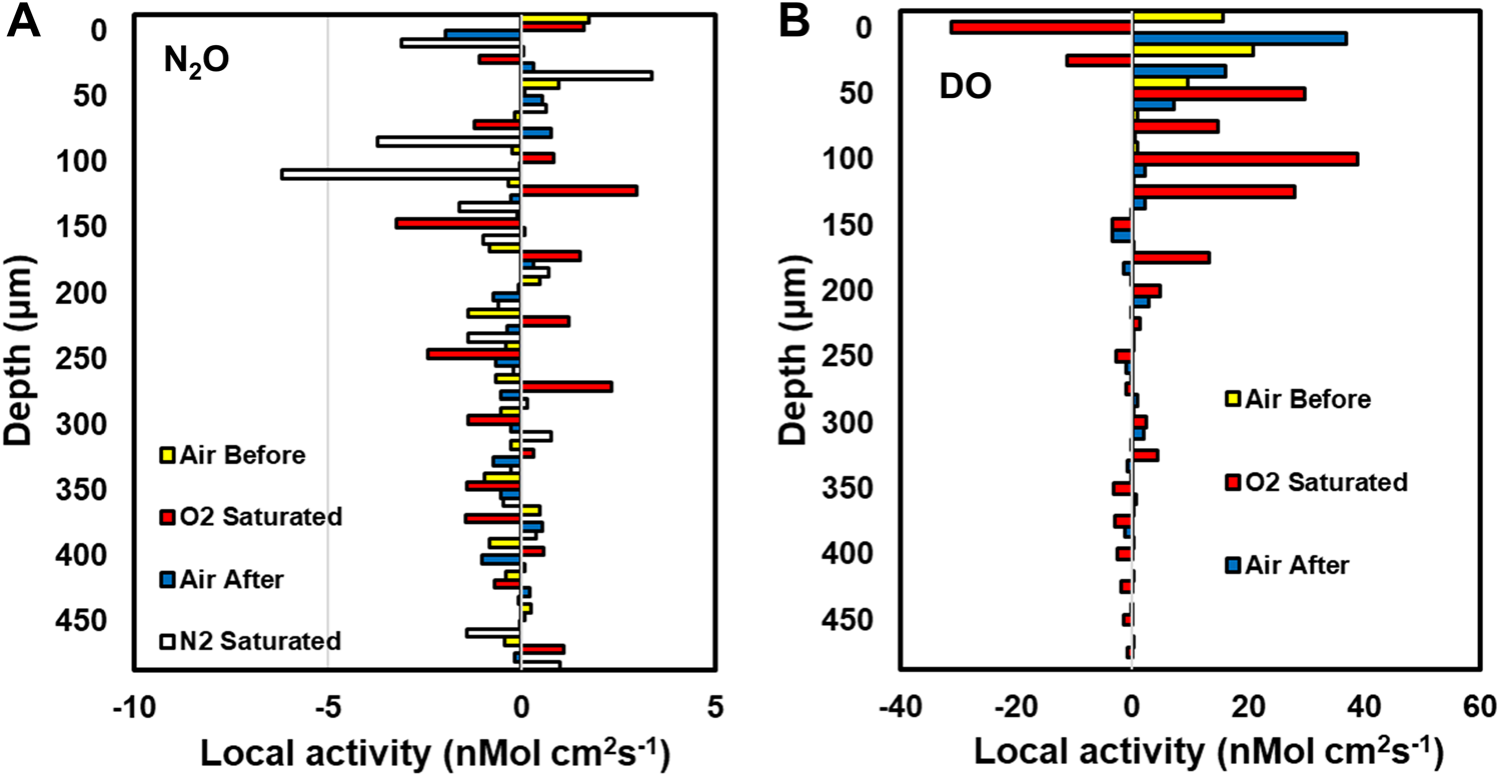
Local aerobic respiration and denitrification activity as a function of depth in a PAO1 biofilm colony under sparging with air (air before O_2_), 10 min after sparging with pure oxygen (O_2_ Saturated), 10 min after returning to sparging with air (air after O_2_) and 80 min after sparging with pure nitrogen (N_2_). (**A**) N_2_O consumption and (**B**) DO consumption. Note, negative consumption indicates production and so the stimulation of N_2_O production under N_2_ sparging indicates high denitrification activity in the top 150 µm 0f the biofilm.

Sparging with pure oxygen resulted in supersaturation of the bluk fluid by approximately a factor of 5 which was consistent with switching from air (21% O_2_) (Fig. 6). This resulted in suppression of denitrification and stimulation of aerobic respiration (Fig. 6A,B respectively). In air the biofilm became anoxic at approximately 100 µm depth but when supersaturated with O_2_ penetration increased so that the conditions became anoxic at 300 µm depth in the biofilm colony. Sparging with pure N_2_ stimulated the production of N2O demonstrating an increase in denitrification (Fig. 6A). Similar to the data shown in Fig. 4 there was local N_2_O production throughout the biofilm colony, although there was a lot of variation wih depth (Fig. 7A). However on sparging with pure nitrogen there was greater N_2_O production in the upper 150 µm of the biofilm. Sparging with air showed most aerobic respiration in the upper 75 µm of the biofilm (Fig. 7B). However, when sparging with pure O_2_ the greatest aerobic respiration activity was depressed to 125 µm depth.

### Influence of tobramycin on aerobic respiration

**Figure 8:**
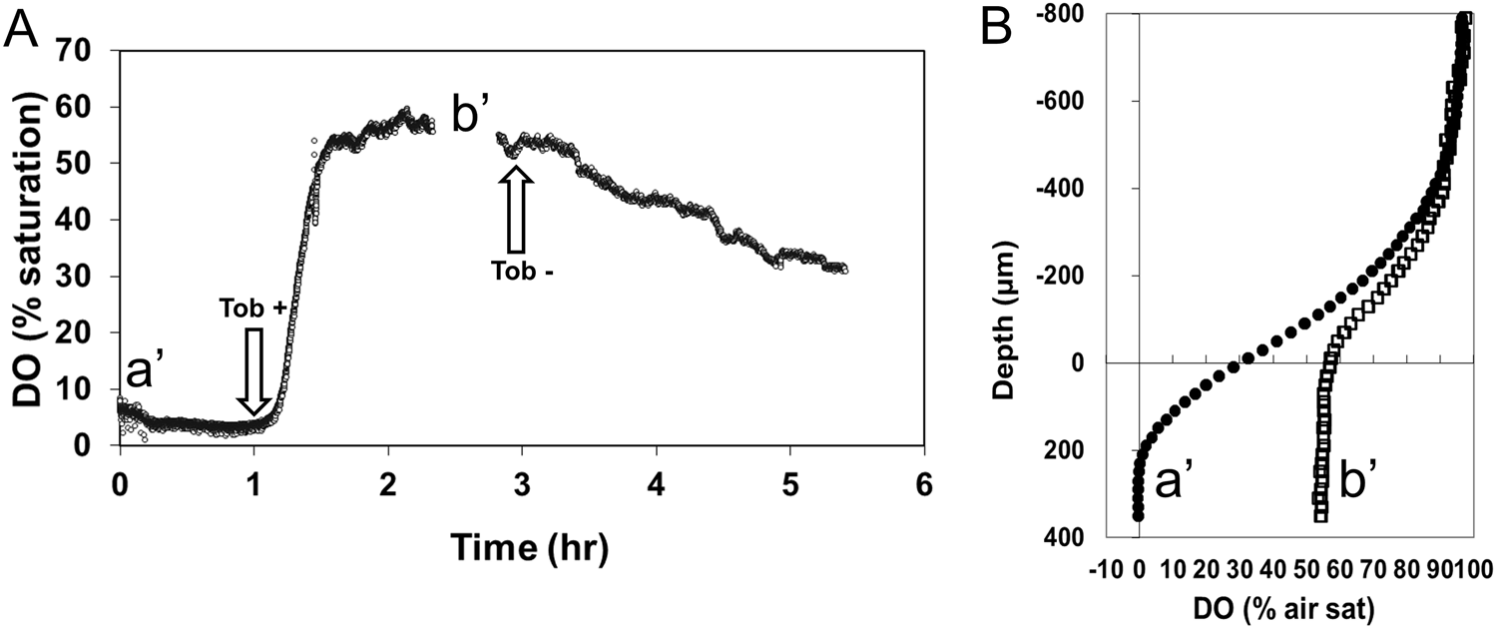
Tobramycin reduced aerobic respiration, but activity immediately recovers after its removal. (**A**) Time trace of DO at 100 µm depth in a PAO1 colony biofilm. Tobramycin was added at 1 h and removed at 1 h after addition. Depth profiles were taken immediately prior to the time trace (a′) and 1.5 h after antibiotic addition (b′). The gap in the time trace is where the second profile was taken. (**B**) DO profiles measured prior to the start of the time trace (a′, black circles) and 1.5 h after (b′, open circles) tobramycin addition.

The response of tobramycin addition colony biofilms was investigated by means of a DO microsensor positioned in the aerobic respiration zone at 100 µm depths inside the colony. Figure 8A shows that during the first hour after starting the experiment, the DO saturation was in steady state and below 10%, indicative for an actively ongoing aerobic respiration. Upon addition of tobramycin, the DO saturation rapidly increased from < 10% to approximately 50% within 0.5 h as less oxygen was consumed by overlying cells allowing greater penetration into the biofilm A steady stated in air saturation at ca. 55% saturation was reached within ca. 1 h after tobramycin addition. However, after tobramycin was removed by changing the medium the DO began to steadily fall as the gradient was reestablished, suggesting that aerobic respiration rapidly recovered.

In addition to the dynamics in aerobic respiration as response to tobramycin addition, Fig. 6B shows the spatial change of the aerobic respiration zone inside the PAO1 colony biofilms. In the absence of tobramycin (Fig. 6B, profile a′), the presence of an active aerobic respiration inside the colony biofilm caused oxygen to deplete in depth up to ca. − 200 µm, indicated by a decreasing air saturation. A profile measured immediately prior to removing the tobramycin (Fig. 6B, profile b′) shows that the air saturation increased up to 55% through the entire colony biofilm body (as oxygen penetrated all the way through the colony) and implies that the aerobic respiration zone moved from − 200 µm depth (profile a′) to the surface of the biofilm (equal to zero depth; profile b′).

## Discussion

We used an agar plate colony biofilm model to investigate the spatial distribution of aerobic respiration and denitrification activity in an ostensibly oxic in vitro environment, i.e., what might be expected in the upper and lower airways. Like other studies we found that aerobic respiration near the surface of the *P. aeruginosa* colony biofilm colony created an anoxic region in the interior^6,25^, in our case at below approximately 100 µm depth. Bacterial denitrification in the lung was originally hypothesized on the basis of the CF lung being hypoxic^8^, due to increased epithelial oxygen consumption in combination with luminal hypoxia due to mucus plugging^26^; however as we, in line with others, show *P. aeruginosa* biofilms can create their own anoxic niches. On the addition of NO_3_^−^ to the bulk liquid we observed an immediate response by the generation of N_2_O. The rapid response suggested that the various enzymes involved in the denitrification steps were already expressed and functional allowing the cells to take immediate advantage of NO_3_^−^ as a terminal electron acceptor. Surprisingly, we saw denitrification occurring even in the outer, oxic, layers of the colony biofilm demonstrating that, in the presence of NO_3_^−^, denitrification is possible even if the local environment is not hypoxic and the biofilm or biofilm aggregates are not large enough to create anoxic regions. This finding is relevant for environments such as the CF lung or chronic wounds where *P. aeruginosa* biofilms consist of aggregates ranging from approximately 10 to 100 µm in diameter^27^, however, it is not clear whether our results would extrapolate to much smaller aggregates. Previously it was assumed that denitrification would only take place in the anoxic zones deep in the biofilm^28^. We used a concentration of 1.2 mM NO_3_^−^, which was relatively high compared to physiological concentrations, however concentrations of approximately 0.8 mM have been reported in sputum from CF patients^29^, the pattern of denitrification distributed throughout the biofilm would be expected to be similar at lower concentrations. We found that although denitrification was occurring throughout the biofilm colony there was a high degree of variation at different depths in the biofilm (Figs. 4 and 6). This finding can be explained by a recent report using mRNA fluorescent in situ hybridization (FISH) reporters for metabolic genes for in situ single cell imaging which showed that in a *P. aeruginosa* biofilm there were single cells to pockets of cells approximately 25 µm in diameter undergoing different metabolic pathways^30^. However, the oxic status of the solution did influence the rates of aerobic respiration and denitrification. When the solution was supersaturated with oxygen denitrification was suppressed and stimulated when made anoix by sparging with nitrogen (Fig. 6). Interestingly, even under oxygen supersaturation the respiration rate was high enough that although oxygen penetration increased from approximately 100 µm depth into the biofilm colony to 250 µm the biofilm was still anoxic at the base (Fig. 6). Oxygen supersaturation did reduce the local respiration rate at the biofilm colony surface was suppressed at the surface and maximum rates occurred at 150 µm depth (Fig. 7). This may be explained by oxygen toxicity at the highest concentration. Under anoxic conditions denitrification was stimulated with the greatest degree of local activity occurring in the upper 125 µm part (Fig. 7). We also found colony to colony variation in the degree of denitrification evidenced by the different amounts of N_2_O generated which ranged between 30 and 120 µM for the different microcolonies and strains (Figs. 3B,D, 7A). Such variation can be explained by the different growth stage of an individual microcolony, as well as strain differences, fluid flow and the nutritional status of the overlying fluid. Thus generalizations on specific rates from one colony or starin to another can not readily be made since these measurements are not conducive for population studies. Although, obviously not identical to the in-situ situation we would like to highlight that our model allows for investigations and manipulations (e.g., antibiotics treatments or varying the local conditions that cannot be carried out in-situ. However, by direct measurement of denitrification and oxygen respiration within the same mucoid and non-mucoid *P. aeruginosa* biofilm colonies at the same time our data supports the conclusions made by Hassett et al.^3^ and Yoon et al.^8^ that denitrification can occurr in the infected CF lung based on transcriptomic profiling.

With respect to antibiotic tolerance generally, it is assumed that part of the mechanism in *P. aeruginosa* biofilm infections, such as found in infected lung and chronic wounds, is due to inactivity within the biofilm due to nutritional limited dormancy. This hypothesis is based largely on experiments conducted in such a manner that nutrient conditions predilect for aerobic respiration. However, in the presence of NO_3_^−^, there is likely activity, albeit lower than that when metabolizing aerobically (i.e. Fig. 5), all the way through the biofilm. After addition of tobramycin we observed an immediate reduction in aerobic respiration, but the microprofiles suggested it was never completely arrested. Furthermore, on removal of the tobramycin, the gradient began to steadily reestablish, suggesting that although there was inhibition there was no complete killing over the short exposure time of one hour. Recently it has been reported that phezanine, which is produced by *P. aeruginosa*, and can promote survival under anoxic conditions, can also provide tolerance to antibiotics^6^ which may explain our results. The decrease in aerobic respiration could also be due to either a general reduction of metabolic activity without killing or by inducing a dispersal event. While we were making the measurements we did not visually observe dispersal of the colony. To draw more definitive conclusions regarding the explanation of the mechanism resulting in the reduced aerobic respiration it would be interesting to measure viability with cell counts or confocal microscopy to assess effects on viability using live/dead staining and which would also detect changes in structure.

### The agar colony biofilm model

Our static biofilm model on agar plates immersed in MSM allowed the set-up of the microelectrodes to achieve simultaneous measurement of DO and N_2_O in close proximity in terms of location and depth in the biofilm was technically challenging. An advantage of using agar-grown colony biofilms was that we limited the probability of breaking the microelectrodes, which is a critical issue with biofilms grown on rigid surfaces such as glass. A disadvantage of using a soft material such as agar was that we could not detect the base of the colony biofilm with the applied sensors at the applied resolution and recording time, and unlike with a rigid surface, gradients developed at the biofilm-agar interface and into the agar as evidenced by the inflection point in N_2_O concentration at approximately 250 µm depth as shown in Fig. 2. Another limitation of the model was that its complexity made it difficult to operate at 37 °C, which is more relevant when discussing our results in the context of infections. It is likely that the profiles would be similar, but conversion rates would be underestimated at room temperature, and DO would be less at 37 °C than room temperature. Historically bacterial biofilms have been studied using experimental models in which biofilms are grown on rigid surfaces immersed in an aqueous solution containing nutrients for growth^31^. However, increasingly soft substrate models are being developed to grow biofilms where biofilms are grown on hydrogel substrates under no, or low, shear to simulate growth on soft tissue such as might be found in an infected lung or chronic wound environment^32^. In some cases, the biofilms are grown directly on a hydrogel infused with nutrients^32,33^ or alternatively on a filter placed on the hydrogel as a “colony biofilm”^34,35^. These biofilms are grown under air rather than being immersed in a liquid. Colonies or lawns grown on agar plates are also increasingly being used as simple biofilm models for mechanical testing either after scraping the colonies off or directly on the colony itself^36–38^. While undoubtedly these colonies have high population densities there is limited information on how well they represent a “classical” biofilm phenotype. Recently Hoiby et al.^39^ presented evidence to suggest that *P. aeruginosa* spread as a lawn on an agar plate switched between a planktonic phenotype to a biofilm phenotype after between 5 and 7 h of incubation, evidenced by tolerance to antibiotics and production of the exopolysaccharide Psl in the extracellular polymeric substance (EPS) matrix. Schiessl et al.^6^ also reported ciprofloxacin tolerance in *P. aeruginosa* colony biofilms and related this to metabolic gradients using microelectrodes to show strong DO and redox gradients with an oxic region at the top of the colony and anoxic conditions at ≤ 50 µm depth. Such gradients are caused by a combination of metabolic activity (consumption and production) and transport limitation into, out of and within the colony. They are well recognized as a characteristic of the biofilm phenotype and can explain phenomena such as the development of microenvironments and antibiotic tolerance through stationary phase-like dormancy to the build-up of cell signaling molecules for quorum sensing co-ordination of the population^40^. Although we recognize that colonies grown on an agar plate and then submerged in liquid differ markedly from conventional biofilms grown on solid surfaces under liquid, we found our model useful to demonstrate that in such colonies strong gradients develop as they do in other biofilms. And importantly that denitrification can occur even in the oxic upper layers of the biofilm simultaneously with aerobic respiration. It is unclear whether there may be pockets of cells in micro-anoxic niches within the oxic layer, which may explain/have implications related to tolerance against antibiotics. Another unresolved aspect is if a small population of cells are denitrifying, despite having access to oxygen as a terminal electron acceptor, which may explain the occurrence of denitrifying ‘persisters/survivors’ in the hypoxic environment that instantly switch on upon availability of oxidative nitrogen species. Nevertheless, our data reveal another aspect of metabolic versatility in *P. aeruginosa* biofilms that may help explain their persistence in chronic infections, and recalcitrance to antibiotic therapy.

## Supplementary Information

Supplementary Figure 1.

Supplementary Figure 2.

Supplementary Figure 3.

Supplementary Figure 4.

Supplementary Legends.
