# Supplementary figures and images for: Nitrate respiration occurs throughout the depth of mucoid and non-mucoid Pseudomonas aeruginosa submerged agar colony biofilms including the oxic zone
Source: Sci Rep. 2022 May 20;12:8557. doi: 10.1038/s41598-022-11957-4 (PMC9123002; doi:10.1038/s41598-022-11957-4)

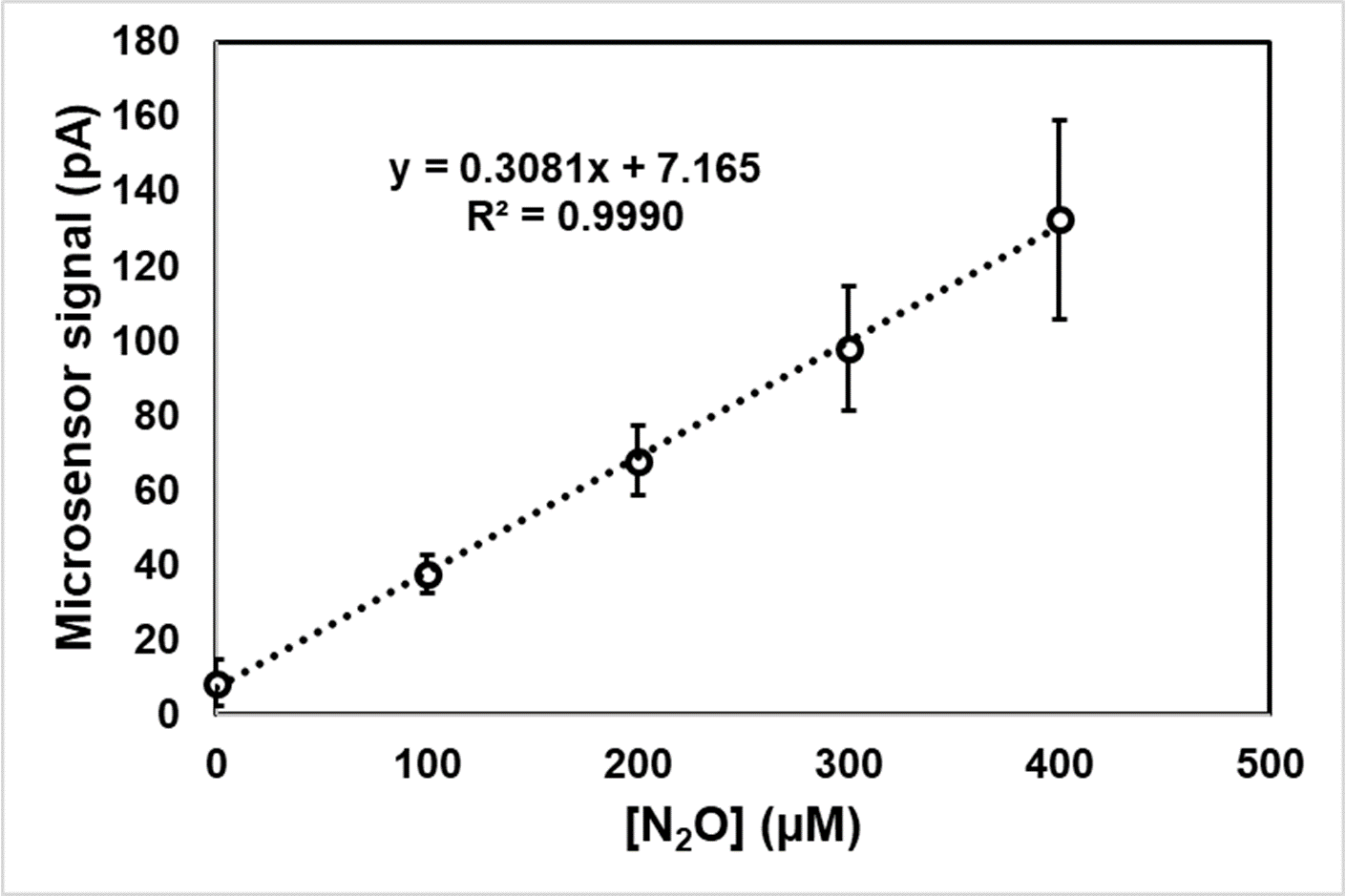

Supplement: Supplementary file 1 — Supplementary Figure 1. [file 41598_2022_11957_MOESM1_ESM.png]

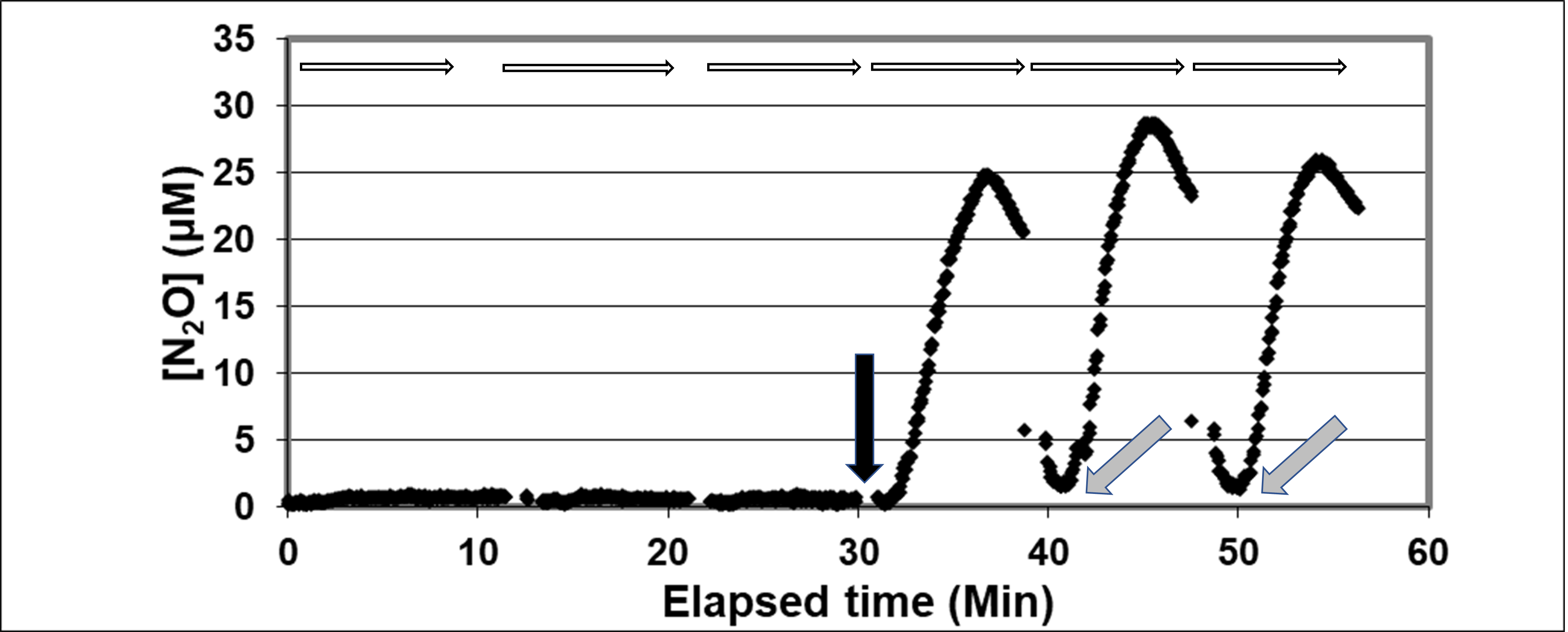

Supplement: Supplementary file 2 — Supplementary Figure 2. [file 41598_2022_11957_MOESM2_ESM.bmp]

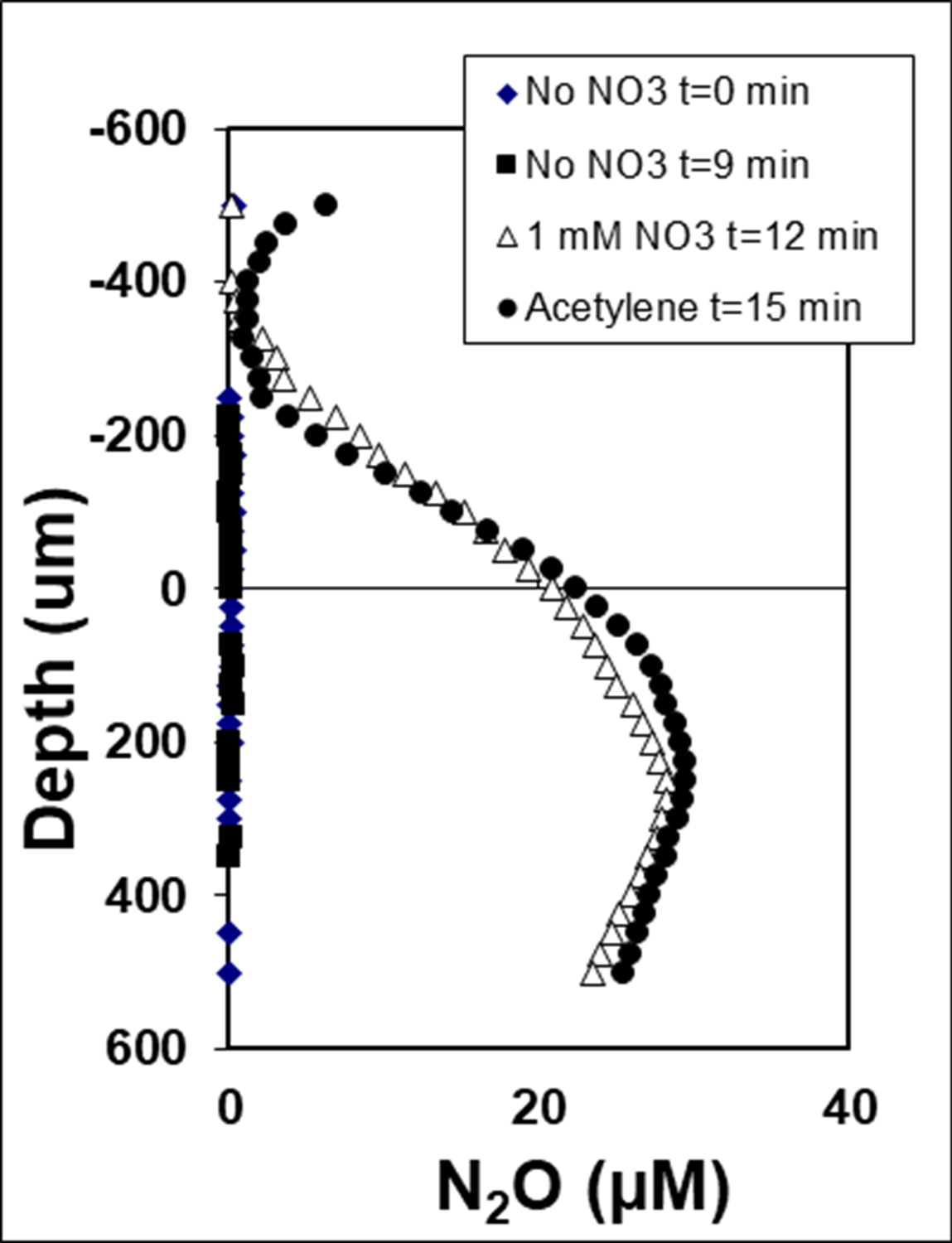

Supplement: Supplementary file 3 — Supplementary Figure 3. [file 41598_2022_11957_MOESM3_ESM.tif]

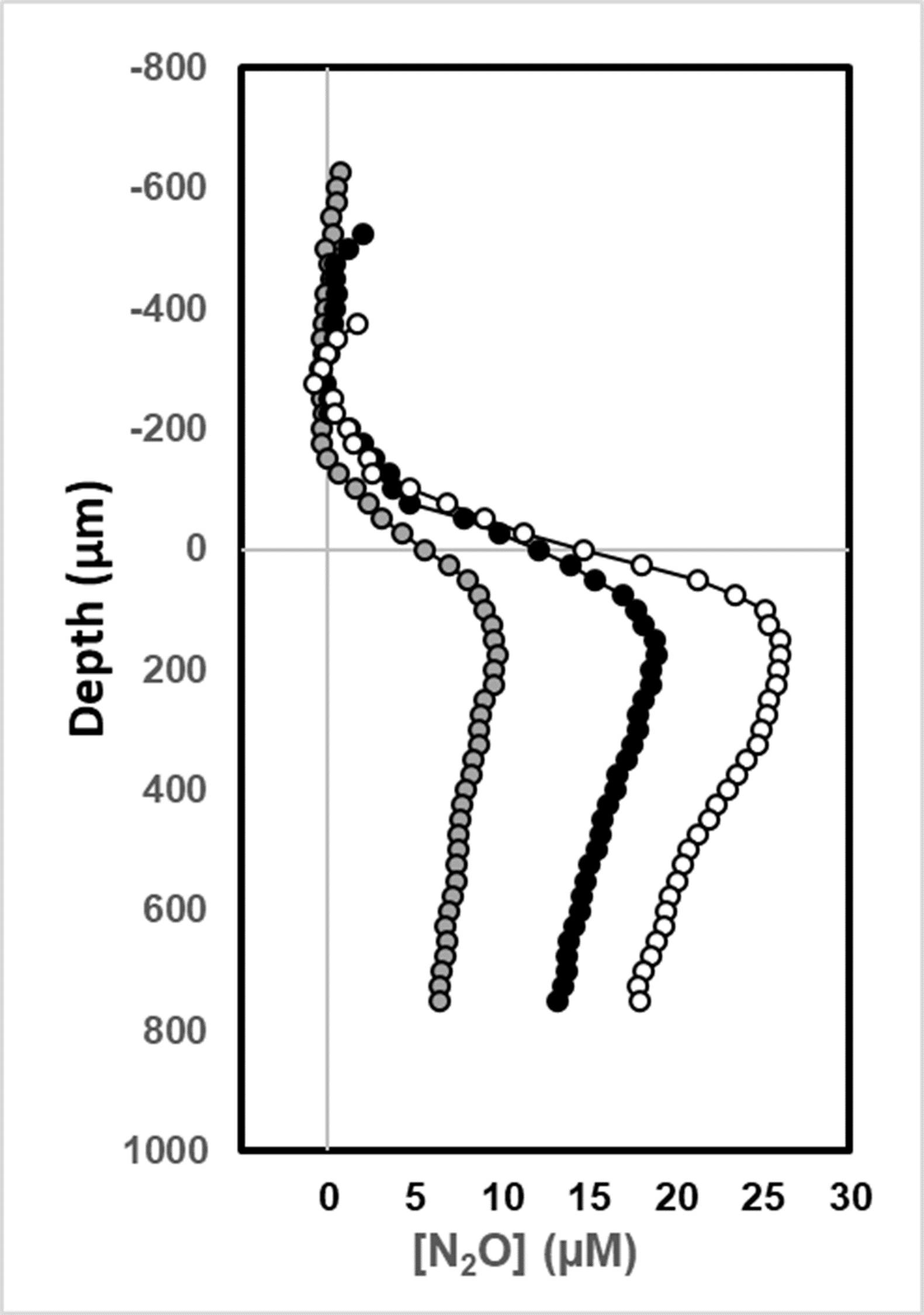

Supplement: Supplementary file 4 — Supplementary Figure 4. [file 41598_2022_11957_MOESM4_ESM.png]
