# Supplementary material for: Nitrate respiration occurs throughout the depth of mucoid and non-mucoid Pseudomonas aeruginosa submerged agar colony biofilms including the oxic zone
Source: Sci Rep. 2022 May 20;12:8557. doi: 10.1038/s41598-022-11957-4 (PMC9123002; doi:10.1038/s41598-022-11957-4)
Supplement: Supplementary file 5 — Supplementary Legends. [file 41598_2022_11957_MOESM5_ESM.docx]

SUPPLEMENTAL DATA

**Supplemental Figure 1.** Calibration of a N_2_O microelectrode in MSM showing the mean of 5 independent curves taken over different days The variation of the slope as expressed as the percent of 1SD of the mean was 21% and the intercept was 117% illustrating that the although the slope was relatively stable there was day to day baseline drift of the signal with zero N_2_O.

Supplemental Figure 2. Time trace during profiling showing the production of N_2_O after the addition of 1.2 mM NO_3_^-^ (black arrow). Three depth profiles were made prior to addition and 3 after addition. The start of the open arrow was where the microelectode was positioned 500 µm above the approxiamte surface of the biofilm colony and the arrowhead end is approxiametly 500 µm below the surface of the biofilm into the underlying agar. There was very little variability in signal prior to adding the nitrate (1SD = 1.07 % of the mean). However, there was baseline drift between the 3 profiles after NO_3_^-^ addition (grey arrows) which could be corrected by subtracting the signal in the bulk liquid where no N_2_O was expected.

**Supplemental Figure 3.** Profiles in a FRD1 colony with no spiked NO_3_^-^ showed no production of N_2_O. After a 1.2 mM NO_3_^-^ spike, N_2_O was immediately produced in the biofilm, as shown by the profile. With the addition of acetylene there was no obvious change in the profile indicating that N_2_O was not being converted to N_2_. The profiles were made from 500 µm until the biofilm-liquid interface (0 µm) to 500 µm depth within the colony in 25 µm incremental steps.

**Supplemental Figure 4.** Profiles in a PAO1 colony with no spiked NO3- showed N_2_O production suggesting there was enough nitrate in the BHI for denitrification under our experimental conditions (grey circles). After a 1.2 mM NO3- spike, N_2_O was immediately stimulated in the biofilm, as shown by the profiles taken at 6 and 31 minutes post spiking (black and open circles respectively). The profiles were made from 500 µm until the biofilm-liquid interface (0 µm) to 500 µm depth within the colony in 25 µm incremental steps.
